# Comparison between calcaneus quantitative ultrasound and the gold standard DXA in the ability to detect osteoporosis in chronic obstructive pulmonary disease patients

**DOI:** 10.1186/s13018-023-04211-8

**Published:** 2023-10-16

**Authors:** Wandee Chanprasertpinyo, Chuchard Punsawad, Rapheeporn Khwanchuea, Naparat Sukkriang, Pirada Yincharoen, Chaiwat Rerkswattavorn

**Affiliations:** School of Medicine, Walailak University, 222, Thai Buri, Tha Sala, Nakhon Si Thammarat, 80160 Thailand

**Keywords:** Chronic obstructive pulmonary disease, Osteoporosis, Quantitative ultrasound

## Abstract

**Background:**

Osteoporosis is a prevalent comorbidity in patients with COPD that is usually underrecognized and hence, undertreated. Compared to the gold standard dual-energy X-ray absorptiometry (DXA), calcaneus quantitative ultrasound (QUS) is less expensive, more portable, and more accessible, especially in less developed countries. The aim of this study was to investigate the ability of calcaneus QUS to screen and prescreen for osteoporosis in patients with COPD.

**Methods:**

This cross-sectional study enrolled 67 males older than 50 years with clinically stable COPD. DXA scans of the lumbar spine (L2–4) and femoral neck were performed. QUS of the right calcaneus (AOS-100) was used to assess the broadband ultrasound attenuation (BUA), speed of sound (SOS), osteo sono-assessment index (OSI), and T-score. When the T-score was ≤  − 2.5, osteoporosis was diagnosed by both DXA and QUS.

**Results:**

Forty-eight patients (71.6%) had DXA T-scores ≤  − 2.5 at either the lumbar spine or femoral neck. All QUS parameters (BUA, SOS, OSI, and T-score) could discriminate DXA-determined osteoporosis (the area under the curve varied from 0.64 to 0.83). The QUS T-score was significantly moderately correlated with the DXA T-score at both the femoral neck (*r* = 0.55) and lumbar spine (*r* = 0.52). The sensitivity and specificity of QUS in identifying osteoporosis were 10.4% and 94.7%, respectively. The positive and negative predictive values were 83.3% and 29.5%, respectively. When a QUS T-score of 0.09 was used as the cutoff, the sensitivity exceeded 90%, and 15% of the DXA scans were not warranted.

**Conclusions:**

The sensitivity and specificity of calcaneus QUS were not sufficient for QUS to be used as an alternative to DXA for osteoporosis screening. However, QUS may be useful for prescreening before DXA to identify COPD patients who have either a high or low likelihood of osteoporosis. Consequently, QUS reduces the need for DXA referral.

## Background

Chronic obstructive pulmonary disease (COPD) is a significant public health problem resulting in socioeconomic burden due to its high prevalence, morbidity, and mortality [1, 2]. Osteoporosis is a significant morbidity in COPD patients that is usually underrecognized and hence undertreated [3]. Patients are typically asymptomatic until fractures occur. Osteoporosis and osteoporosis fractures are extremely common in COPD patients. The overall prevalence of osteoporosis in COPD patients, according to previous studies, ranges from 14 to 66%. The significant associated factors include being underweight (body mass index (BMI) < 18.5 kg/m^2^), the presence of sarcopenia, an older age, a high Global Initiative for Chronic Obstructive Lung Disease (GOLD) stage, a low physical activity level, vitamin D deficiency, a low fat-free mass index (FFMI) and the use of systemic steroids [4, 5].

Vertebral compression fractures (VCFs) are the most common type of osteoporosis-induced fractures and are common in COPD patients, with a prevalence ranging from 24 to 79% [6]. VCFs can cause kyphosis and subsequently result in impaired lung function in COPD patients. Moreover, osteoporosis fractures are associated with pain and lead to decreased mobility, impairing individuals’ ability to perform activities of daily living (ADL) and quality of life [7, 8]. Therefore, the early detection of osteoporosis is considerably essential for the management of patients with COPD.

The gold standard for the diagnosis of osteoporosis is the measurement of bone mass density (BMD) using dual-energy X-ray absorptiometry (DXA). However, the accessibility of DXA is limited due to the increasing demand for bone health assessments with population aging. Thus, there is a long waiting time, which can delay diagnosis and treatment in patients with osteoporosis. Moreover, DXA is relatively costly and involves a low level of ionizing radiation.

Quantitative ultrasound (QUS) is an attractive method for bone health assessments and has gained much attention in recent years. It is portable and inexpensive, does not emit ionizing radiation, and is more accessible to the public, particularly in less developed countries [9]. The calcaneus is the only site recommended by the International Society of Clinical Densitometry (ISCD) for QUS [10]. A recent meta-analysis demonstrated that calcaneus QUS can potentially be used as a prescreening tool for the assessment of osteoporosis [11]. The role of QUS in screening for osteoporosis in COPD patients has not been studied.

Therefore, the aims of this study were to investigate (1) the prevalence of osteoporosis and factors associated with osteoporosis, (2) the ability of calcaneus QUS to identify COPD patients with DXA-determined osteoporosis and (3) investigate the suitability of QUS as a prescreening tool for osteoporosis in COPD patients.

## Methods

### Study design and population

This is a cross-sectional study that was conducted from March 2019 to April 2020. The study was approved by the Human Research Ethics Committee of Walailak University, with certification ID number WUEC-19-059-01. We recruited patients with a known diagnosis of COPD from the outpatient COPD clinic at Thasala Hospital, a primary care hospital located in the rural area of Nakhon Si Thammarat, Thailand. Patients were considered eligible for this study if they met the following inclusion criteria: were male, were older than 50 years old, and had clinically stable COPD confirmed by post-bronchodilator FEV1/FVC < 0.7, where clinically stable was defined as the absence of exacerbation in the previous four weeks. Patients were excluded if they had one or more of the following criteria: were diagnosed with osteoporosis or taking anti-osteoporosis drugs, including calcium or vitamin D supplements; had asthma, a rheumatic disease, chronic liver or renal disease, primary or secondary hyperparathyroidism, thyroid dysfunction, or Cushing syndrome; or had taken oral corticosteroids in the preceding six weeks. Informed consent was obtained from all enrolled patients before any study-related procedures were performed.

### Measurements

Demographic data were collected by interviewing the patients and reviewing their medical records. An internist from the researcher team interviewed the patients and collected the following data: smoking history, symptom assessment using the COPD Assessment Test (CAT), and physical activity level using the Global Physical Activity Questionnaire (GPAQ). CAT contains an eight-item questionnaire including the severity of cough, sputum production, chest tightness, dyspnea, limited activities, confidence leaving home, sleeplessness, and energy. The score for each item ranges from 0 to 5. A higher CAT score indicates poorer health status [12].

The patients’ medical records were reviewed to determine their current and previous medications, spirometry values, history of hospitalization due to COPD exacerbation in the past 12 months, and comorbidities. The comorbidities were summarized by the Charlson comorbidity index [13]. The Charlson comorbidity index used in this study consisted of 17 items corresponding to different medical comorbid conditions. The total score is the summation of each comorbidity item with different clinical weights. The higher scores indicate a greater mortality risk and more severe comorbid conditions [14]. The severity of airflow limitation was graded using post-bronchodilator % of predicted FEV1 values and categorized using The Global Initiative for Chronic Obstructive Lung Disease (GOLD) system into GOLD grade I (FEV1 ≥ 80% predicted), grade II (FEV1 50–79% predicted), grade III (FEV1 30–49% predicted), or grade IV (FEV1 < 30% predicted) [15].

Osteoporosis was determined by two methods at the same visit for all patients. First, DXA scans (Osteosys Dexxum T, OsteoSys, Korea) of the second to the fourth lumbar vertebrae (L2–4) and the right femoral neck were taken. Second, a QUS of the right calcaneus was performed using the Acoustic Osteo-Screener ultrasound device (AOS-100, Aloka Co., Ltd., Japan). QUS measures the following parameters: speed of sound (SOS), broadband ultrasound attenuation (BUA), osteo sono-assessment index (OSI), and T-score. SOS (m/s) is the ultrasound velocity to the heel. The OSI is calculated using the following formula: OSI ꞊ TI × SOS^2^. TI is a value that is proposed to be related to BUA. T-score is obtained by comparing the patient’s OSI with the mean OSI value obtained from young, healthy Japanese adults aged 20–44 [16, 17].

According to the WHO, osteoporosis is present when BMD is 2.5 standard deviations (SDs) or more below the average value for normal young adults (a T-score of ≤  − 2.5 SD). Osteopenia was recorded when the T-score was between − 1.0 SD and − 2.5 SD. T-scores higher than − 1.0 SD were considered normal (a T-score ≥ − 1) [18]. Patients with an abnormal DXA T-score were referred to an orthopedist for appropriate treatment. In this study, we used the DXA T-score criteria to interpret the QUS T-score.

We measured BMI and fat-free mass with bioelectrical impedance analysis (TANITA SC-330, Tanita Corp., Japan). The FFMI was calculated as the FFM divided by the square of the patient’s height. BMI was categorized as low (< 18.5 kg/m^2^), normal (18.5–24.9 kg/m^2^), overweight (25.0–29.9 kg/m^2^), or obese (≥ 30 kg/m^2^) [19]. FFMI was considered to be depleted when FFMI < 16 kg/m^2^ in the men [20]. A blood sample was drawn and stored at −80 °C in a freezer for subsequent biochemical analysis. Serum 25-hydroxyvitamin D [25(OH)D] was measured by an enzyme-linked immunosorbent assay (ELISA) kit (ab213966 25-OH Vitamin D ELISA kit, Abcam, UK).

### Statistical analysis

Statistical Package for Social Sciences (SPSS), version 26, and STATA, version 14.1, were used for data analyses. The descriptive statistics are presented as frequencies with percentages and means ± SDs or medians with interquartile ranges (IQRs). Independent T-tests and Chi-square tests were used to compare the continuous and categorical variables, respectively. For nonparametric analysis, the Mann–Whitney U test was used. Logistic regression was performed to identify the significant factors associated with osteoporosis. Spearman’s rank correlation was performed to assess the correlation between the DXA T-score and calcaneus QUS T-score. Receiver operating characteristic (ROC) analysis was performed to (1) calculate the areas under the curves (AUCs) to compare the ability of each calcaneus QUS parameter with that of DXA to detect osteoporosis, and (2) identify the appropriate calcaneus QUS T-score cutoff for diagnosing osteoporosis with high sensitivity.

## Results

### Patient characteristics

Among the 89 patients who met all the inclusion and exclusion criteria, 67 patients responded to participate in this study. A comparison of the demographic characteristics of the osteoporotic and nonosteoporotic groups is summarized in Table 1. The mean age of the whole cohort was 69.6 years, and 52% of the patients were older than 70 years. The participants in the osteoporotic group were slightly older than those in the nonosteoporotic group, but the difference was not significant. Almost all patients (94%) had used inhaled corticosteroids in the past year. The duration and dose of inhaled corticosteroids did not significantly differ between the two groups. According to the GOLD classification system of airflow limitation severity, most of the patients in the two groups were GOLD grade II. The median CAT score, number of exacerbations in the previous year, and number of oral corticosteroid users did not differ between the two groups. The mean BMI of all the participants was 21.27 ± 3.8 kg/m^2^, and the majority of the patients (73.1%) had a BMI ≥ 18.5 kg/m^2^. The mean FFMI of all participants was 17.35 ± 1.92 kg/m^2^, and 18 patients (26.9%) had a depleted FFMI (FFMI < 16 kg/m^2^). The mean BMI and FFMI values were significantly lower in the osteoporotic group than in the nonosteoporotic group. All calcaneus parameters other than BUA were significantly lower in the osteoporotic group. In both groups, the femoral T-score was lower than the lumbar spine T-score.

**Table 1:** Baseline characteristics of the study population

	Osteoporosis *N* = 48	Nonosteoporosis *N* = 19	*p*-value
Age, years	70.21 ± 8.97	68 ± 7.62	0.348
Body mass index (kg/m^2^)	20.35 ± 3.62	23.62 ± 3.26	0.001*
Tobacco use			
Current smoker, no (%)	8 (16.7%)	5 (26.3%)	0.496
Ex-smoker, no (%)	39 (81.3%)	13 (68.4%)	
Pack-years	28.50 (10, 60)	30 (7.5, 40)	
Charlson comorbidity index	3.73 ± 1.14	3.53 ± 1.12	0.513
Duration of inhaled corticosteroid, year	4.42 (1.25, 7.63)	2.75 (1, 7)	0.587
Fluticasone equivalent dose, mcg/day	500 (250, 500)	250 (250, 500)	0.744
Oral corticosteroid uses in the past year, no (%)	17 (35.4%)	6 (31.6%)	0.766
GOLD grade, no (%)			
I: FEV_1_ ≥ 80% predicted	7 (14.6%)	4 (21.1%)	0.289
II: FEV_1_ 50–79% predicted	21 (43.8%)	10 (52.6%)	
III: FEV_1_ 30–49% predicted	12 (25%)	5 (26.3%)	
IV: FEV_1_ < 30% predicted	8 (16.7%)	0 (0%)	
COPD Assessment Test (CAT™) score	12 (4, 18)	11 (1, 16)	0.411
Numbers of exacerbation in the previous year	0 (0, 1)	0 (0, 1)	0.514
FFMI (kg/m^2^)	16.94 ± 1.95	18.38 ± 1.42	0.002*
Global Physical Activity Questionnaire total activity, metabolic equivalent intensity min/week	840 (380, 1920)	840 (280, 2520)	0.850
Serum 25(OH)D level, ng/ml	7.80 ± 0.37	7.86 ± 0.28	0.531
Calcaneus QUS			
SOS, m/s	1533.15 ± 17.97	1553.47 ± 25.79	0.004*
BUA, dB/MHz	53.38 ± 18.18	60.76 ± 18.22	0.140
OSI	2.41 ± 0.30	2.76 ± 0.67	0.040*
T-score	−1.51 ± 0.88	−0.48 ± 1.55	0.012*
Femoral neck T-score	−3.49 ± 1.04	−1.37 ± 1.06	< 0.001*
Lumbar spine T-score	−3.21 ± 1.33	−0.63 ± 1.50	< 0.001*

Values are mean ± SD or median (range).

*FEV1* force expiratory volume in the first second, *BMI* body mass index, *FFMI* fat-free mass index, *SABA* short-acting beta-agonists, *SAMA* short acting muscarinic antagonists, *LAMA* long-acting muscarinic antagonist, *25(OH)D* 25-hydroxyvitamin D

*p value is significant

### Prevalence of osteoporosis determined by DXA and factors associated with osteoporosis

The mean T-score and prevalence of osteoporosis and osteopenia are presented in Table 2. The mean T-score of the femoral neck (−2.89 ± 1.42) was significantly lower than that of the lumbar spine (−2.48 ± 1.8, *p* = 0.002). The prevalence of osteoporosis and that of osteopenia at the femoral neck were 64.2% and 28.4%, respectively. The prevalence of osteoporosis and that of osteopenia at the lumbar spine were 50.7% and 34.3%, respectively. When the lowest T-score at either the lumbar spine or femoral neck was used for diagnosis, there were 48 patients (71.6%) with osteoporosis and 16 patients (23.9%) with osteopenia.

**Table 2:** Mean T-score and prevalence of osteoporosis and osteopenia

Measurement	Mean T-score (SD)	Osteoporosis *n* (%)	Osteopenia *n* (%)	Normal *n* (%)
DXA				
Femoral neck	−2.89 (1.42)	43 (64.2)	19 (28.4)	5 (7.5)
Lumbar spine	−2.48 (1.80)	34 (50.7)	23 (34.3)	10 (14.9)
Calcaneus QUS	−1.22 (1.19)	6 (9.0)	39 (58.2)	22 (32.8)

Osteopenia: T-score between −1.0 SD and −2.5 SD, Osteoporosis: T-score <  − 2.5 SD

The significant risk factors associated with osteoporosis were BMI and FFMI, as shown in Table 3. The COPD patients with a low BMI (BMI < 18.5 kg/m^2^) had an almost ninefold increased risk of osteoporosis than did the patients with a normal BMI. The patients who had an FFMI < 17 kg/m^2^ had a five times higher risk of osteoporosis than did those with an FFMI greater than 17 kg/m^2^.

**Table 3:** Factors associated with osteoporosis

Variable	OR	95% CI	*p*-value
Age (years)			
50–59^a^	Reference		
60–69	1.43	(0.30, 6.74)	0.652
70–79	1.14	(0.26, 5.09)	0.861
≥ 80	5.71	(0.52, 62.66)	0.154
BMI (kg/m^2^)	0.77	(0.64, 0.91)	0.003*
Underweight (BMI < 18.5)	8.84	(1.06, 74.03)	0.044*
Normal weight (BMI 18.5–24.9^a^)	Reference		
Overweight (BMI ≥ 25)	0.62	(0.16, 2.44)	0.498
Tobacco use (Pack-years)	1.01	(0.99, 1.03)	0.359
Charlson comorbidity index	1.18	(0.73, 1.91)	0.507
Inhaled corticosteroid uses in the past year	0.83	(0.08, 8.55)	0.878
Oral corticosteroid uses in the past year	1.19	(0.38, 3.69)	0.766
GOLD grade			
Grade I^a^	Reference		
Grade II	1.20	(0.28, 5.07)	0.804
Grade III	1.37	(0.27, 6.87)	0.701
Grade IV	1.00	(0, 1.00)	0.999
COPD Assessment Test (CAT™) score	1.02	(0.96, 1.09)	0.469
Numbers of exacerbation in previous year	1.00	(0.86, 1.17)	0.952
FFMI (kg/m^2^)	0.63	(0.44, 0.89)	0.009*
< 17	4.91	(1.26, 19.06)	0.022*
≥ 17^a^	Reference		

*OR* odd ratio, *CI* confidence interval

^a^Reference category

*p value is significant

### Ability of calcaneus QUS to detect osteoporosis

**Fig. 1:**
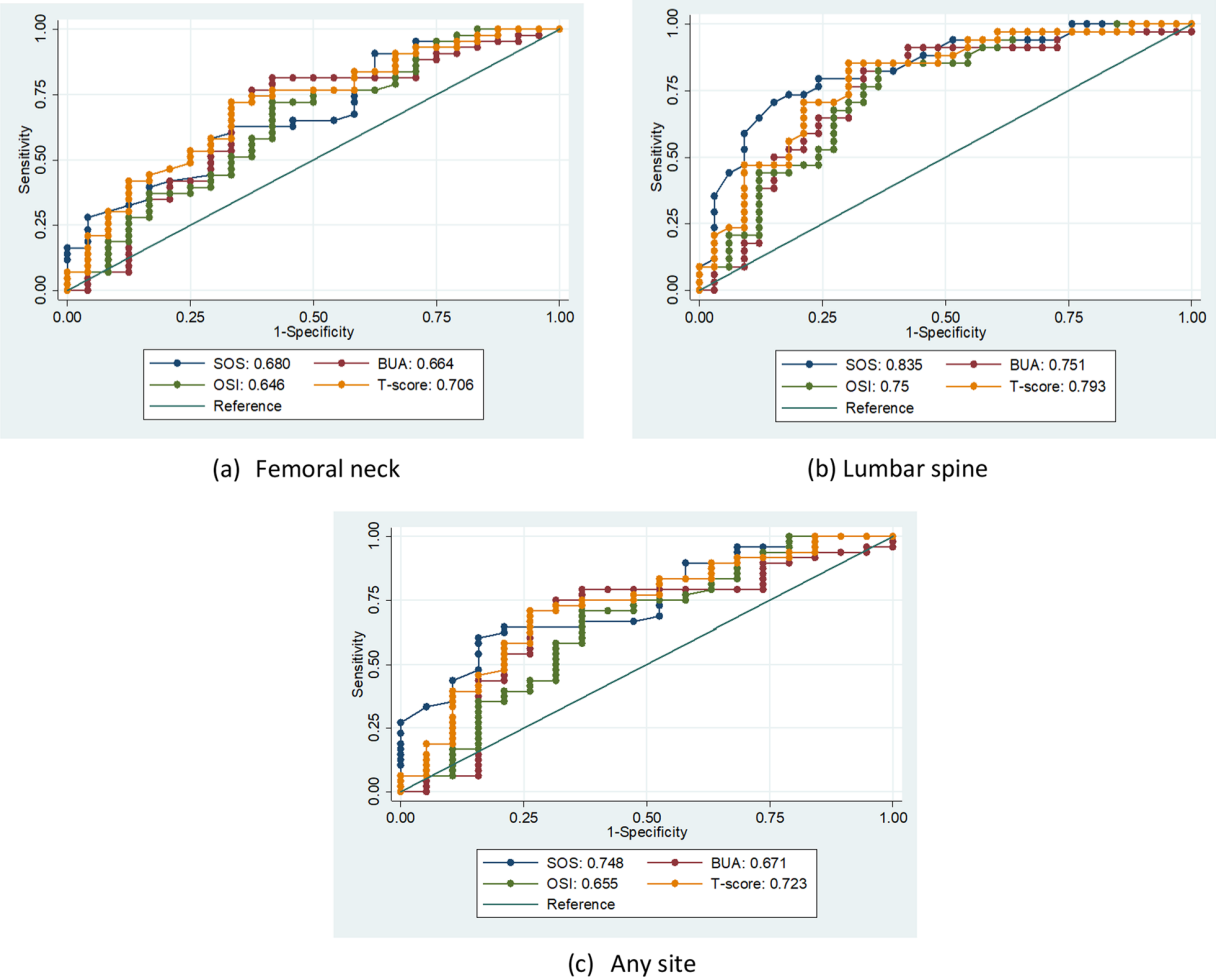
Receiver operating characteristics (ROC) curves for each QUS parameter (BUA, SOS, OSI, T-score) for detecting osteoporosis at **a** Femoral neck, **b** lumbar spine, and **c** Any site (defined as T-score ≤ −2.5 at either femoral neck or lumbar spine)

The mean T-score determined using calcaneus QUS was −1.22 ± 1.19, which was significantly higher than the T-score determined using DXA at both the femoral neck and lumbar spine (*p* < 0.001) (Table 2). The calcaneus QUS T-score was significantly correlated with the DXA T-score at the femoral neck (*r* = 0.55, *p* < 0.001) and lumbar spine (*r* = 0.52, *p* < 0.001). Each QUS parameter was analyzed using ROC analysis to determine which QUS parameter was able to identify the participants with osteoporosis at the femoral neck, the lumbar spine, and either site (either the femoral neck or the lumbar spine). All four QUS parameters could discriminate osteoporosis at both sites, with an AUC ranging from 0.64 to 0.83 and *p* < 0.05, indicating statistical significance (see Fig. 1). The AUC reflects the accuracy of the test. In general, an AUC closer to 1.0 indicates a more accurate test, while an AUC of 0.5 suggests the test has no discriminative ability. The accuracy of the QUS T-score was in an acceptable range to detect osteoporosis at either site (AUC 0.7–0.79).

Among all four QUS parameters, the T-score is commonly used in clinical practice by most physicians. In this study, we interpreted the result of the QUS T-score on the basis of the DXA T-score criteria. Using a DXA T-score ≤ −2.5 as a reference for diagnosing osteoporosis, the prevalence of osteoporosis detected by calcaneus QUS was only 9%, which was significantly lower than that detected by DXA at both the femoral neck and lumbar spine (*p* < 0.001). The sensitivity, specificity, positive predictive value, and negative predictive value of the different QUS T-score cutoff values are presented in Table 4. When a QUS T-score ≤ −2.5 was used as the cutoff, the sensitivity was less than 15%, while the specificity was approximately 95%. The positive predictive value was 83.3%, and the negative predictive value was approximately 29.5–52.4%. Therefore, subjects with a QUS T-score ≤ −2.5 had a high likelihood of osteoporosis. However, with this cutoff level, calcaneus QUS missed diagnosing approximately 90% of the patients who were diagnosed with osteoporosis by DXA. When the QUS T-score cutoff value was increased, the sensitivity increased, while the specificity decreased. When the sensitivity exceeded 80%, the specificity was less than 70% (Table 4).

**Table 4:** The ability of QUS T-score at the different cutoff to predict DXA-determined osteoporosis

Site	QUS T-score*	Sensitivity (%)	Specificity (%)	PPV (%)	NPV (%)
Any site	−2.50	10.42	94.73	83.33	29.50
	−1.25	70.83	73.68	87.18	50.00
	−0.80	83.33	47.37	80.00	52.94
	−0.09	91.66	31.57	77.19	60.00
Femoral neck	−2.50	11.62	95.83	83.33	37.70
	−1.25	72.09	66.67	79.49	57.14
	−0.80	83.72	41.67	72.00	58.82
	−0.09	93.02	29.17	70.18	70.00
Lumbar spine	−2.50	14.70	96.97	83.33	52.46
	−1.25	85.29	69.70	74.36	82.14
	−0.88	91.18	45.45	63.27	83.33
	−0.09	97.06	27.27	57.89	90.00

*PPV* positive predictive value, *NPV* negative predictive value

^*^The threshold value of QUS T-score below this point, osteoporosis is diagnosed by calcaneus QUS

### Suitability of QUS as a prescreening tool for osteoporosis

To screen for osteoporosis, tests with high sensitivity are required. We used ROC analysis to identify the QUS T-score cutoff value at which the sensitivity exceeded 90%. When the cutoff value exceeded −2.5, the sensitivity increased. The optimal cutoff value for the QUS T-score to detect osteoporosis was 0.88 for lumbar osteoporosis and −0.09 for femoral neck osteoporosis, with a sensitivity exceeding 90% (Table 4). When a QUS T-score cutoff of −0.88 was used, QUS detected lumbar osteoporosis with a sensitivity of 91.18% and a specificity of 45.45%. The misclassification rate was 4.47%. Approximately 26% of the DXA scans were not warranted. When a QUS T-score of −0.09 was used, the sensitivity and specificity of QUS to detect osteoporosis at either site and at the femoral neck were approximately 92% and 30%, respectively. DXA was not warranted in 14.9% of the cases, and the misclassification rate was approximately 5%.

## Discussion

In the present study, the prevalence of osteoporosis determined by the gold standard DXA was 71.6%, which was much higher than that previously reported for the Thai male population (12%) [21]. The results from previous studies conducted in different countries using DXA showed that the prevalence of osteoporosis in COPD patients varied from 14 to 66% [5]. The significant factors associated with osteoporosis in this study were BMI < 18.5 kg/m^2^ (OR = 8.84) and FFMI < 17 kg/m^2^ (OR = 0.63). A meta-analysis of two studies also demonstrated a similar result: having a BMI < 18.5 increased the risk of osteoporosis by 4.26-fold [4, 22, 23]. FFMI (FFMI < 16 kg/m^2^) was a risk factor associated with osteoporosis in COPD patients in two studies [24, 25]. We found that FFMI < 17 kg/m^2^, even when it was not defined as a depleted FFMI, increased the risk of osteoporosis in COPD patients. Regardless of the risk factors for osteoporosis, COPD patients have an approximately 2.8 times higher risk of osteoporosis than do those without COPD. Therefore, patients with COPD should be screened for osteoporosis [4].

According to the Thai Osteoporosis Foundation (TOPF), the BMD of males should be measured by DXA after the age of 70 [26]. In this study, 52% of the male patients were older than 70 years. Another possible indication for a BMD assessment in COPD patients is prolonged glucocorticoid use (prednisolone 7.5 mg/day for at least three consecutive months). The patients in this study were prescribed glucocorticoids when they had an acute exacerbation, and they did not take the glucocorticoids for more than three months. Therefore, most COPD patients in this study had two main issues regarding DXA: (1) they had no indication for DXA measurements, and (2) DXA machines are scarce in rural areas and are usually available at tertiary care hospitals in large cities. As a result, COPD patients living in rural areas may have difficulty traveling to a city, especially if they live far from a city. Being old and experiencing breathlessness in COPD patients are also limitations to traveling long distances. The cost also increases when patients must pay for transportation. We believe that COPD patients in other countries also have similarly limited accessibility to DXA. Therefore, the calcaneus QUS was investigated in this study to determine (1) its ability to identify COPD patients with DXA-determined osteoporosis and (2) its suitability as a prescreening tool for osteoporosis in COPD patients.

There are four calcaneus QUS parameters (SOS, BUA, OSI, and T-score). The present study demonstrated that all the calcaneus QUS parameters can identify osteoporosis in COPD patients, with significant AUCs ranging from 0.646 (for OSI) to 0.835 (for SOS). An AUC greater than 0.7 reflects an acceptable level of accuracy of QUS. The AUCs of all the QUS parameters in this study determined using the AOS-100 device were comparable to those reported in previous studies using QUS devices from other manufacturers [27, 28]. SOS had the highest AUC compared to the other QUS parameters in discriminating osteoporosis at any site and the lumbar spine. We found a moderate correlation between the QUS T-score and DXA T-score at both the femoral neck and spine. The degree of correlation was higher for the femoral neck, which was consistent with the result in a previous study [29].

In this study, we compared the DXA T-score with the QUS T-score because few studies have used the AOS-100 device, and a previous study that used this device assessed the QUS T-score with respect to the DXA T-score [30]. When the DXA T-score cutoff value was used for diagnosis, in this study, the prevalence of osteoporosis and that of osteopenia determined using calcaneus QUS were 6% and 58.2%, respectively, which were significantly lower than the prevalence determined using the gold standard DXA. The results from previous studies in COPD patients that used calcaneus QUS devices made by other manufacturers and interpreted the QUS T-score with respect to the DXA T-score showed that the prevalence of osteoporosis varied from 8.7 to 35% and the prevalence of osteopenia varied from 35 to 51.35% [24, 31, 32]. However, previous studies did not compare the accuracy of QUS to that of DXA. The present study is the first to explore the utility of calcaneus QUS compared to that of DXA for detecting osteoporosis in COPD patients.

We found that when a T-score ≤ −2.5 was used as the cutoff for diagnosing osteoporosis, calcaneus QUS had an extremely low sensitivity for detecting DXA-defined osteoporosis. The negative predictive value was relatively low. As a result, most of the subjects classified as nonosteoporotic on the basis of the QUS T-score (QUS T-score > −2.5) additionally needed to undergo a DXA scan to confirm the absence of osteoporosis. Because the QUS T-score cutoff value differs by the device used, there is no single cutoff value that can be applied without errors in the number of COPD patients diagnosed with osteoporosis. Many studies also agree that the DXA T-score cutoff value cannot be used as the QUS T-score cutoff value [10, 11]. Different QUS devices yield different results. When a T-score threshold of ≤ −2.5 was used for different QUS devices, the prevalence of osteoporosis varied by more than tenfold [10]. Different QUS devices also demonstrated different sensitivities, ranging from 34.8 to 88.4%, and specificities, ranging from 41.2 to 91.9%, when a QUS T-score cutoff of −2.5 was used [11, 33, 34]. The QUS measurement also varies by the skeletal site [28]. Therefore, calcaneal QUS cannot replace DXA for the diagnosis of osteoporosis according to the WHO classification.

However, many studies have demonstrated that calcaneus QUS may be used as a prescreening tool before DXA [11, 28, 35]. With a device-specific upper threshold of 90% sensitivity and a lower threshold of 90% specificity, individuals who have either a high or low likelihood of osteoporosis can be identified [10, 36]. In this cohort of COPD patients, when a QUS T-score of −0.09 was used as the cutoff, the patients with a QUS T-score at or above this threshold were considered to have a low likelihood of having osteoporosis. As a result, 15% of the DXA scans were not warranted. In addition, patients who had a QUS T-score below the 0.09 cutoff could undergo an early pharmacological intervention consisting of calcium and vitamin D supplementation while waiting for the DXA scan. The cutoff used in this study is limited to male COPD patients undergoing scans with the AOS-100 device. Due to the variety of QUS devices and cutoffs, calcaneus QUS prescreening algorithms must be based on a device-specific cutoff that has been validated in the populations for whom they are intended to be used [11]. Due to the high prevalence of osteoporosis among COPD patients, COPD patients should be screened for osteoporosis, but DXA is not widely available. We believe that the use of QUS as a prescreening tool could be useful in several aspects. First, COPD patients who meet the DXA measurement criteria but do not want to travel to the city for DXA scans may find it useful. Physicians may be able to use the QUS information to determine the necessity for DXA referral. Second, even COPD patients who do not meet the criteria for DXA assessments are at risk of osteoporosis and should be screened. QUS could serve as a prescreening tool for these patients.

This study has several limitations. First, the sample size was relatively small for determine an appropriate cutoff that achieves sufficient statistical power [11]. Second, the investigated device in this study has been used in fewer previous studies than have other QUS devices. Third, the results of this study were specific to male COPD patients in Thailand. Additional studies are needed to establish appropriate cutoffs for females and other ethnic groups.

## Conclusions

The ability of calcaneus QUS to serve as an alternative diagnostic tool to DXA is limited by the lack of consensus on which QUS device and diagnostic cutoff is best. However, this study demonstrated that calcaneus QUS can serve as a useful prescreening tool for osteoporosis in COPD patients. It can reduce the DXA referral rate by 15%. Future studies in a larger cohort of COPD patients are needed to identify appropriate device-specific cutoffs for a prescreening algorithm.

## Data Availability

The datasets used and/or analyzed during the current study are available from the corresponding author on reasonable request.

## Abbreviations

COPD: Chronic obstructive pulmonary disease
FEV1: Force expiratory volume in the first second
FVC: Force vital capacity
BMI: Body mass index
BMD: Bone mass density
FFMI: Fat free mass index
25(OH)D: 25-Hydroxyvitamin D
DXA: Dual-energy X-ray Absorptiometry
QUS: Quantitative ultrasound
SOS: Speed of sound
BUA: Broadband ultrasound attenuation
OSI: Osteo sono-assessment index

## References

[1] Adeloye D, Chua S, Lee C, Basquill C, Papana A, Theodoratou E (2015). Global and regional estimates of COPD prevalence: Systematic review and meta-analysis. J Glob Health.

[2] Singh D, Agusti A, Anzueto A, Barnes PJ, Bourbeau J, Celli BR, et al. Global strategy for the diagnosis, management, and prevention of chronic obstructive lung disease: the GOLD science committee report 2019. Eur Respir J. 2019;53(5). 10.1183/13993003.00164-2019 30846476

[3] Okazaki R, Watanabe R, Inoue D (2016). Osteoporosis associated with chronic obstructive pulmonary disease. J Bone Metab.

[4] Chen YW, Ramsook AH, Coxson HO, Bon J, Reid WD (2019). Prevalence and risk factors for osteoporosis in individuals With COPD: a systematic review and meta-analysis. Chest.

[5] Bitar AN, Syed Sulaiman SA, Ali IAH, Khan I, Khan AH (2019). Osteoporosis among patients with chronic obstructive pulmonary disease: systematic review and meta-analysis of prevalence, severity, and therapeutic outcomes. J Pharm Bioallied Sci.

[6] Inoue D, Watanabe R, Okazaki R (2016). COPD and osteoporosis: links, risks, and treatment challenges. Int J Chron Obstruct Pulmon Dis.

[7] Sarkar M, Bhardwaj R, Madabhavi I, Khatana J (2015). Osteoporosis in chronic obstructive pulmonary disease. Clin Med Insights Circ Respir Pulm Med.

[8] Migliorini F, Giorgino R, Hildebrand F, Spiezia F, Peretti GM, Alessandri-Bonetti M, et al. Fragility fractures: risk factors and management in the elderly. Medicina (Kaunas, Lithuania). 2021;57(10). 10.3390/medicina57101119 PMC8538459 34684156

[9] Chin K-Y, Ima-Nirwana S (2013). Calcaneal quantitative ultrasound as a determinant of bone health status: what properties of bone does it reflect?. Int J Med Sci.

[10] Krieg MA, Barkmann R, Gonnelli S, Stewart A, Bauer DC, Del Rio BL (2008). Quantitative ultrasound in the management of osteoporosis: the 2007 ISCD official positions. J Clin Densitometry.

[11] Thomsen K, Jepsen DB, Matzen L, Hermann AP, Masud T, Ryg J (2015). Is calcaneal quantitative ultrasound useful as a prescreen stratification tool for osteoporosis?. Osteoporosis Int.

[12] Pisi R, Aiello M, Calzetta L, Frizzelli A, Tzani P, Bertorelli G (2023). The COPD assessment test and the modified Medical Research Council scale are not equivalent when related to the maximal exercise capacity in COPD patients. Pulmonology.

[13] Charlson ME, Pompei P, Ales KL, MacKenzie CR (1987). A new method of classifying prognostic comorbidity in longitudinal studies: development and validation. J Chronic Dis.

[14] Deyo RA, Cherkin DC, Ciol MA (1992). Adapting a clinical comorbidity index for use with ICD-9-CM administrative databases. J Clin Epidemiol.

[15] Alvar A, Bartolome RC, Gerard JC, David H, Antonio A, Peter B (2023). Global initiative for chronic obstructive lung disease 2023 report: GOLD executive summary. Eur Respir J.

[16] Tsuboi S, Hayakawa T, Kanda H, Fukushima T (2009). The relationship between clustering health-promoting components of lifestyle and bone status among middle-aged women in a general population. Environ Health Prev Med.

[17] Tsuda-Futami E, Hans D, Njeh C, Fuerst T, Fan B, Li J (1999). An evaluation of a new gel-coupled ultrasound device for the quantitative assessment of bone. Br J Radiol.

[18] Prevention WHOSGot, Management of O. Prevention and management of osteoporosis : report of a WHO scientific group. Geneva: World Health Organization; 2003.

[19] Expert Panel on the Identification E, Overweight To, Adults Oi. Executive summary of the clinical guidelines on the identification, evaluation, and treatment of overweight and obesity in adults. Arch Internal Med. 1998;158(17):1855–67. 10.1001/archinte.158.17.1855 9759681

[20] Baarends EM, Schols AM, Mostert R, Wouters EF (1997). Peak exercise response in relation to tissue depletion in patients with chronic obstructive pulmonary disease. Eur Respir J.

[21] Pongchaiyakul C, Apinyanurag C, Soontrapa S, Soontrapa S, Pongchaiyakul C, Nguyen T, et al. Prevalence of osteoporosis in Thai men. J Med Assoc Thailand = Chotmaihet thangphaet. 2006;89:160–9. 16579001

[22] Ogura-Tomomatsu H, Asano K, Tomomatsu K, Miyata J, Ohmori N, Kodama M, et al. Predictors of Osteoporosis and Vertebral Fractures in Patients Presenting with Moderate-to-Severe Chronic Obstructive Lung Disease. COPD: J Chronic Obstruct Pulmonary Dis. 2012;9(4):332–7. 10.3109/15412555.2012.667850 22489911

[23] Hattiholi J, Gaude GS (2014). Prevalence and correlates of osteoporosis in chronic obstructive pulmonary disease patients in India. Lung India.

[24] Vrieze A, de Greef MHG, Wýkstra PJ, Wempe JB (2007). Low bone mineral density in COPD patients related to worse lung function, low weight and decreased fat-free mass. Osteoporos Int.

[25] Graat-Verboom L, Spruit MA, van den Borne BE, Smeenk FW, Martens EJ, Lunde R (2009). Correlates of osteoporosis in chronic obstructive pulmonary disease: an underestimated systemic component. Respir Med.

[26] Songpatanasilp T, Sritara C, Kittisomprayoonkul W, Chaiumnuay S, Nimitphong H, Charatcharoenwitthaya N (2016). Thai Osteoporosis Foundation (TOPF) position statements on management of osteoporosis. Osteoporosis Sarcopenia.

[27] Oral A, Esmaeilzadeh S, Yalıman A, Sindel D, Kürsüz Köseoğlu P, Aydın T (2019). The ability of calcaneal and multisite quantitative ultrasound variables in the identification of osteoporosis in women and men. Turkish J Phys Med Rehabil.

[28] Steiner B, Dimai HP, Steiner H, Cirar S, Fahrleitner-Pammer A (2019). Prescreening for osteoporosis with quantitative ultrasound in postmenopausal white women. J Ultrasound Med.

[29] Cetin A, Ertürk H, Celiker R, Sivri A, Hasçelik Z (2001). The role of quantitative ultrasound in predicting osteoporosis defined by dual X-ray absorptiometry. Rheumatol Int.

[30] Chang H-C, Hsieh C-F, Lin Y-C, Tantoh DM, Ko P-C, Kung Y-Y, et al. Does coffee drinking have beneficial effects on bone health of Taiwanese adults? A longitudinal study. BMC Public Health. 2018;18(1):1273. 10.1186/s12889-018-6168-0 PMC6245613 30453911

[31] Ramachandran K, Mani SK, Gopal GK, Rangasami S. Prevalence of bone mineral density abnormalities and factors affecting bone density in patients with chronic obstructive pulmonary disease in a Tertiary Care Hospital in Southern India. J Clin Diagn Res. 2016;10(9):OC32–OC4. 10.7860/JCDR/2016/22464.8551 PMC5071990 27790490

[32] Bhattacharyya P, Paul R, Ghosh M, Dey R, Dey R, Barooah N (2011). Prevalence of osteoporosis and osteopenia in advanced chronic obstructive pulmonary disease patients. Lung India.

[33] Díez-Pérez A, Marín F, Vila J, Abizanda M, Cervera A, Carbonell C (2003). Evaluation of calcaneal quantitative ultrasound in a primary care setting as a screening tool for osteoporosis in postmenopausal women. J Clin Densitometry.

[34] Felder M, Haldemann R, Anderhub HP (2000). Value of ultrasound study and dual energy x-ray absorptiometry (DEXA) for assessment of risk of osteoporosis. Praxis.

[35] Fitzgerald GE, Anachebe T, McCarroll KG, O’Shea F. Calcaneal quantitative ultrasound has a role in out ruling low bone mineral density in axial spondyloarthropathy. Clin Rheumatol. 2020. 10.1007/s10067-019-04876-9 31953568

[36] Clowes JA, Peel NF, Eastell R (2006). Device-specific thresholds to diagnose osteoporosis at the proximal femur: an approach to interpreting peripheral bone measurements in clinical practice. Osteoporosis Int.

