# Correction: Comparison between calcaneus quantitative ultrasound and the gold standard DXA in the ability to detect osteoporosis in chronic obstructive pulmonary disease patients

**DOI:** 10.1186/s13018-023-04353-9

**Published:** 2023-11-15

**Authors:** Wandee Chanprasertpinyo, Chuchard Punsawad, Rapheeporn Khwanchuea, Naparat Sukkriang, Pirada Yincharoen, Chaiwat Rerkswattavorn

**Affiliations:** School of Medicine, Walailak University, 222, Thai Buri, Tha Sala, Nakhon Si Thammarat, 80160 Thailand

**Correction: Journal of Orthopaedic Surgery and Research (2023) 18:778** **https://doi.org/10.1186/s13018-023-04211-8**

Following publication of the original article [1], the author reported that the authors were omitted from the author group. Wandee Chanprasertpinyo^1^, Chuchard Punsawad^1^, Rapheeporn Khwanchuea^1^, Naparat Sukkriang^1^, Pirada Yincharoen^1^ and Chaiwat Rerkswattavorn^1*^ have been added to the author group and are presented correctly in this correction article.

First author: Wandee Chanprasertpinyo

Second author: Chuchard Punsawad

Third author: Rapheeporn Khwanchuea

Fourth author: Naparat Sukkriang

Fifth author: Pirada Yincharoen

Corresponding author: Chaiwat Rerkswattavorn

The affiliation of all authors is School of Medicine, Walailak University, 222, Thai Buri, Tha Sala, Nakhon Si Thammarat, 80160, Thailand.

The original article [1] has been corrected.
